# Bone Tumor Environment as a Potential Therapeutic Target in Ewing Sarcoma

**DOI:** 10.3389/fonc.2015.00279

**Published:** 2015-12-23

**Authors:** Françoise Redini, Dominique Heymann

**Affiliations:** ^1^INSERM UMR_S 957, Nantes, France; ^2^Equipe labellisée Ligue contre le Cancer 2012, Nantes, France; ^3^Laboratoire de Physiopathologie de la Résorption osseuse et Thérapie des tumeurs osseuses primitives, Faculté de Médecine, Nantes, France; ^4^CHU Hôtel-Dieu, Nantes, France

**Keywords:** Ewing sarcoma, bone remodeling, bisphosphonate, RANKL, microenvironment, tumor bone niche, 3D models

## Abstract

Ewing sarcoma is the second most common pediatric bone tumor, with three cases per million worldwide. In clinical terms, Ewing sarcoma is an aggressive, rapidly fatal malignancy that mainly develops not only in osseous sites (85%) but also in extra-skeletal soft tissue. It spreads naturally to the lungs, bones, and bone marrow with poor prognosis in the two latter cases. Bone lesions from primary or secondary (metastases) tumors are characterized by extensive bone remodeling, more often due to osteolysis. Osteoclast activation and subsequent bone resorption are responsible for the clinical features of bone tumors, including pain, vertebral collapse, and spinal cord compression. Based on the “vicious cycle” concept of tumor cells and bone resorbing cells, drugs, which target osteoclasts, may be promising agents as adjuvant setting for treating bone tumors, including Ewing sarcoma. There is also increasing evidence that cellular and molecular protagonists present in the bone microenvironment play a part in establishing a favorable “niche” for tumor initiation and progression. The purpose of this review is to discuss the potential therapeutic value of drugs targeting the bone tumor microenvironment in Ewing sarcoma. The first part of the review will focus on targeting the bone resorbing function of osteoclasts by means of bisphosphonates or drugs blocking the pro-resorbing cytokine receptor activator of NF-kappa B ligand. Second, the role of this peculiar hypoxic microenvironment will be discussed in the context of resistance to chemotherapy, escape from the immune system, or neo-angiogenesis. Therapeutic interventions based on these specificities could be then proposed in the context of Ewing sarcoma.

## Introduction

### Ewing Sarcoma: A Clinical Presentation

**Figure 1:**
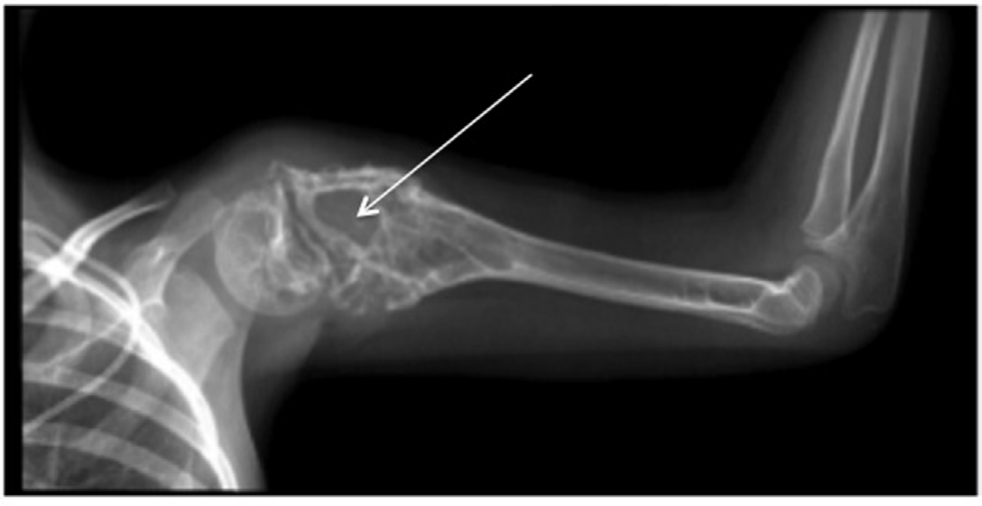
**X-ray of typical severe osteolytic lesions in a Ewing sarcoma patient (arrows: severe osteolytic lesions)**.

Ewing sarcoma was first described by James Ewing in 1921. It is a high-grade neoplasm, and it is the second most common primary bone malignancy in both children and adolescents (1). With peak incidence at 15 years, this disease accounts for 2% of childhood cancers (2). Ewing sarcoma is defined as a bone tumor, which may occur at any site within the skeleton but preferentially affects the trunk and the diaphysis of long bones (2). However, it may occur in extra-skeletal soft tissue in 15% of cases. It is characterized by rapid tumor growth and extensive bone destruction (Figure 1) that can result in bone pain and pathological fracture (3). At the histological level, Ewing sarcoma appears as small, poorly differentiated, round tumor cells positive for the transmembrane glycoprotein CD99 staining (4).

The molecular event that initiates the Ewing’s family of tumors is a typical chromosomal translocation that occurs in cells of mesenchymal origin and that fuses the *EWS* gene on chromosome 22q12 to a member of the erythroblast transformation sequence (ETS) transcription gene family, most commonly *FLI-1*, on 11q24 in 85% of cases (5–7). This translocation leads to the production of the oncogenic fusion gene *EWS-FLI1*, an aberrant transcription factor that promotes tumorigenicity (8, 9). The presence of this fusion gene, which represents the Ewing sarcoma signature, is used as a specific diagnostic marker of the Ewing’s family of tumors thanks to fluorescence *in situ* hybridization and RT-qPCR (10). Numerous biological pathways, such as those involving insulin-like growth factor receptor (IGFR), platelet-derived growth factor receptor (PDGFR), vascular endothelial growth factor receptor (VEGFR), Sonic HedgeHog (SHH) pathway activation, Wnt, and transforming growth factor (TGF)-β receptor II pathway inhibition, are modulated by EWS-FLI1 activity, leading to proliferation, angiogenesis, immune system escape, metastatic potential, and treatment resistance that contribute to the Ewing sarcoma malignant phenotype (11).

### Therapeutic Limits

The on-going treatments for Ewing sarcoma patients are effective in more than 70% of patients with localized disease. They elicit clinical responses in patients with metastatic disease but are not curative due to acquired resistance. Before the 1970s, amputation was the main therapeutic option, with 5-year survival of <20%. The introduction of first radiation and then chemotherapy in the 70s has modified the prognostic significantly, with the 5-year event-free survival rate for localized tumors at around 65%, and the overall survival rate close to 75%. However, the survival rates decrease to 15–25% when metastases are detected at diagnosis, or in patients presenting resistance to treatment or with relapsed disease. In the past three decades, conventional therapies seem to have attained a survival plateau for these metastatic patients (12).

Improved poly-chemotherapy has made it possible to limit surgery and salvage limb, but in about 20% of cases, bone sarcomas have already disseminated at the time of diagnosis. In most cases, the distant metastases are located in the lungs, followed by the skeleton. Although Ewing sarcoma patients with lung metastases have overall survival of 45% at 5 years, those with bone or bone marrow metastases have very poor prognosis, with <25% overall survival at 5 years. In the past, when therapy was limited to local control (surgery), nearly all patients who initially appeared to have a localized tumor developed distant metastases (13). Ewing sarcoma thus needs to be considered as a systemic disease, requiring systemic treatment, i.e., combination chemotherapy, as a rule. However, systemic therapy can never replace definitive local control with surgery and/or radiotherapy. The therapy used for Ewing sarcoma therefore requires a combination of surgery or radiotherapy for localized control and high-intensity chemotherapy for localized and disseminated disease. The most recent protocol for Ewing tumors was the European Ewing tumor Working Initiative of National Groups 99 protocol (EuroEWING99, clinicaltrials.gov no. NCT00020566), which tested the benefits of a different chemotherapy combination involving vincristine, ifosfamide, doxorubicin, and etoposide (VIDE). The protocol was composed of six sequences of VIDE treatment followed by surgery when possible. The histological response to chemotherapy was then evaluated and patients were divided into three arms depending on the localization of the tumor at diagnosis, the volume for unresected tumors, and the percentage of residual cells after treatment. The R1 arm included patients with localized disease and a good response to chemotherapy (<10% of residual cells) or with a volume of <200 ml. The R2 arm included patients with lung metastases and patients with localized tumors and a poor response to chemotherapy, or with a volume of more than 200 ml. Finally, the R3 arm included patients with bone, bone marrow, or multifocal metastases. The current survival rate for EuroEWING patients has attained 80% for localized disease of small volume (R1). Unfortunately, the 5-year survival rate for patients with metastases detected at diagnosis remains around 25% and even around 10% when relapse occurs within the first 2 years following treatment.

The current protocol for Ewing sarcoma patients is the EuroEWING2012 (clinicaltrials.gov no. NCT00987636), which started in December 2014 in Great Britain, with two randomizations: the first compares two chemotherapy protocols (with surgery and/or radiotherapy) and the second randomizes patients with or without bisphosphonate zoledronate (zometa^®^).

Given that survival rates had not evolved in more than three decades, especially for metastatic patients with a very poor initial prognosis, there was an urgent need to define new therapeutic targets for Ewing sarcoma patients. In addition to the tumor cells themselves, targeting the bone tumor microenvironment appears to be promising.

### The Bone Microenvironment Is a Favorable “Niche” for Tumor Progression in Bone

Recently, there has been a dramatic increase in the importance given to the theory that the bone microenvironment participates in determining the “bone niche” in the progression of bone tumors, and in establishing resistance processes to conventional therapies. The concept of “bone niche” is well-recognized in the context of hematological malignancies, such as leukemia (14) or multiple myeloma (15). The “niche” is a functional microenvironment able to both promote the emergence of cancer stem cells and provide all factors required for their development. However, the bone niche is composed of numerous cell types (pre-osteoclasts, pre-osteoblasts, endothelial cells, macrophages, etc.) that are located in the bone matrix, and their functional coordination is a pre-requisite for maintaining the bone and bone niche microarchitecture.

Much research has been published on the role played by the bone microenvironment in establishing metastases in these organs, especially from breast or prostate carcinomas. The concept of bone niche is also currently under discussion in the case of solid tumors, and strengthens the “seed and soil” theory proposed by Paget in 1887, in which tumor cells (“seeds”) colonize receptive foci (“soil”) (16). These data are supported by the fact that specific molecules (such as cadherin and osteopontin) play a part in stabilizing cancer cells in bone niches, mimicking the cell interactions that take place during hemopoiesis, as identified in the pre-metastatic niche in breast carcinoma (17, 18). In addition, carcinoma cells grow well in bone, which stores a variety of cytokines and growth factors, and thus provides an extremely fertile environment for growing tumor cells (19, 20).

The “seed and soil” theory can be also envisaged for primary bone tumors, as tumor growth and metastasis often require constant interactions between tumor cells and their surrounding microenvironment (21–25). This hypothesis has been largely documented in the case of osteosarcoma (26, 27) and chondrosarcoma (28), but very little information is currently available for Ewing sarcoma.

### The Concept of the Vicious Cycle in Ewing Sarcoma

**Figure 2:**
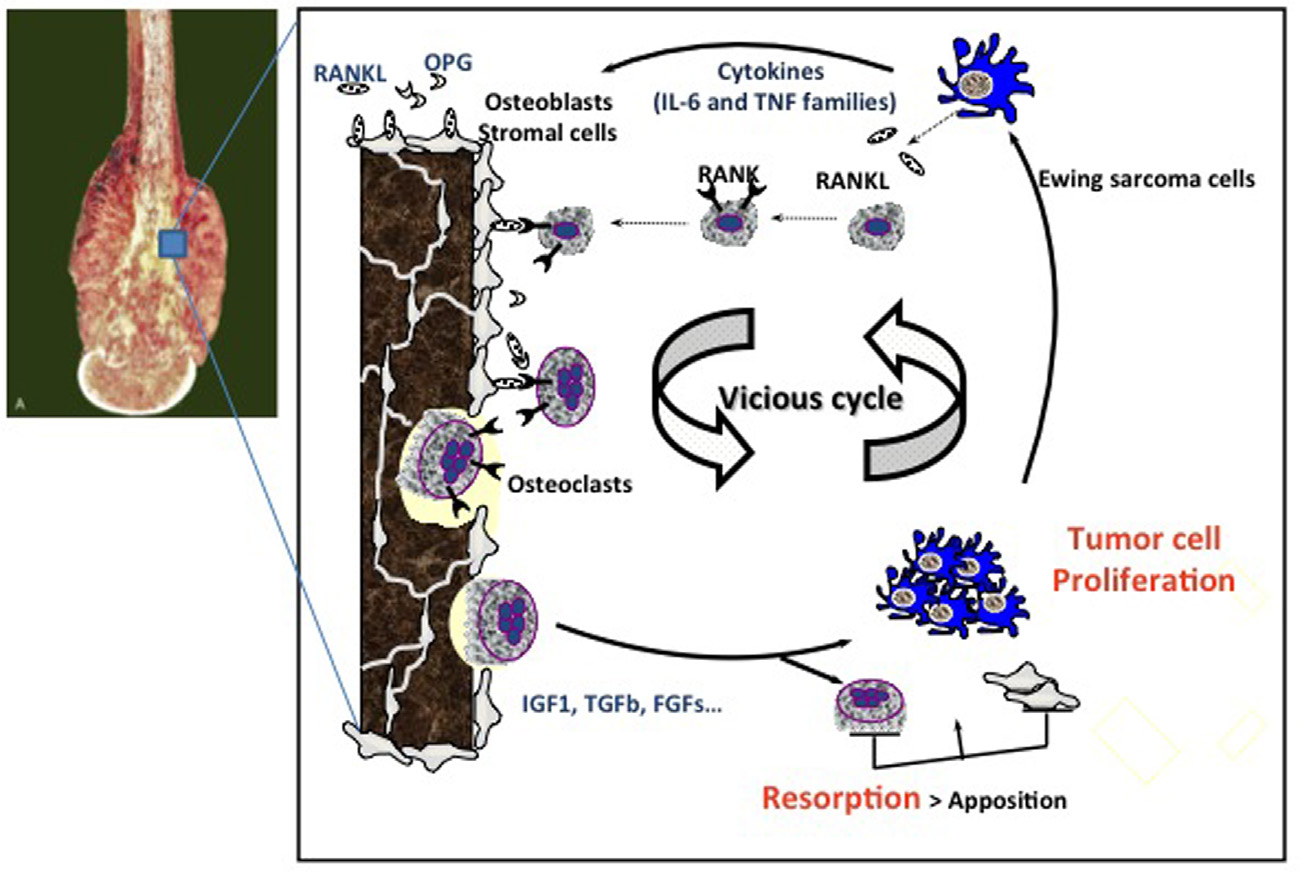
**Vicious cycle between Ewing sarcoma cell proliferation and osteoclast activation**. Tumor cells produce osteoclast activating factors (IL-6, TNF-α, etc.) that will induce osteoclast differentiation and activation. When they resorb bone, osteoclasts allow the release of growth factors stored in the bone matrix, such as IGF-1, FGFs, and TGF-β, which in turn activate tumor cell proliferation. This is the theory of the so-called “vicious cycle.” The molecular OPG/RANKL/RANK triad plays a pivotal role in the regulation of bone resorption. OPG and RANKL are produced by osteoblasts and/or stromal cells, whereas RANK is expressed at the surface of osteoclasts and their precursors. OPG, osteoprotegerin; IL-6, interleukin-6; TNF, tumor necrosis factor; RANK, receptor activator of NF-kB; RANKL, RANK-ligand; IGF1, insulin-like growth factor1; TGF-β, transforming growth factor-β; FGFs, fibroblast growth factors.

Ewing sarcoma is characterized by extensive bone destruction, mainly due to osteolysis (Figure 1). Because Ewing sarcoma cells cannot directly degrade bone, osteoclast activation and subsequent bone resorption may be responsible for the clinical features of bone destruction in this pathology (3). Bone degradation is controlled by osteoclasts, whose differentiation and activation are mainly mediated by receptor activator of NF-kappa B ligand (RANKL), a member of the tumor necrosis factor (TNF) super-family (TNFSF11) after it binds to its receptor RANK expressed at the surface of mature osteoclasts and osteoclast precursors (29) (Figure 2). Osteoprotegerin (OPG) acts as a decoy receptor inhibiting osteoclast formation, function, and survival by preventing the binding of RANKL to its receptor RANK (26).

Interaction between tumor cells, tumor-derived humoral factors, and the bone marrow in the bone niche has been shown to be essential for bone tumor initiation and promotion (30, 31). Targeting the bone microenvironment, and particularly osteoclast activation, may therefore be a promising adjuvant strategy for treating bone tumors, including Ewing sarcoma. The vicious cycle between osteoclasts, bone stromal cells/osteoblasts, and cancer cells has been hypothesized during the progression of primary bone tumors (32) (Figure 2). Tumor cells produce osteoclast activating factors, such as interleukin (IL)-6, TNF-α, or ParaThyroid Hormone-related Peptide (PTH-rP), which induce osteoclast differentiation and activation. When osteoclasts resorb bone, they allow the release of growth factors stored in the bone matrix (TGF-β, IGF-1, PDGF, etc.), which in turn activate tumor cell proliferation (32). Accordingly, inhibiting osteoclast activity is a promising approach for breaking the vicious cycle, and thus indirectly limiting local cancer growth.

In addition, new therapeutic options targeting hypoxia, angiogenesis, bone cells, or mediators in the particular bone microenvironment have been studied extensively at the preclinical level, with the more promising now being proposed in clinical trials. This review will describe the most recent developments in such therapeutic options for Ewing sarcoma patients.

## Targeting Bone Cells in Ewing Sarcoma

Therapeutic agents that target the bone environment and modulate bone metabolism have been studied in preclinical models of primary bone sarcomas, demonstrating a certain degree of efficacy in both osteosarcoma and Ewing sarcoma. Two main strategies are currently being developed: (i) the first directly targets osteoclasts (differentiation, activation, and functions), mainly using bisphosphonates (BPs), and (ii) the second targets the cytokine RANKL, the pivotal cytokine for regulating osteoclast activation.

### Bisphosphonates

**Figure 3:**
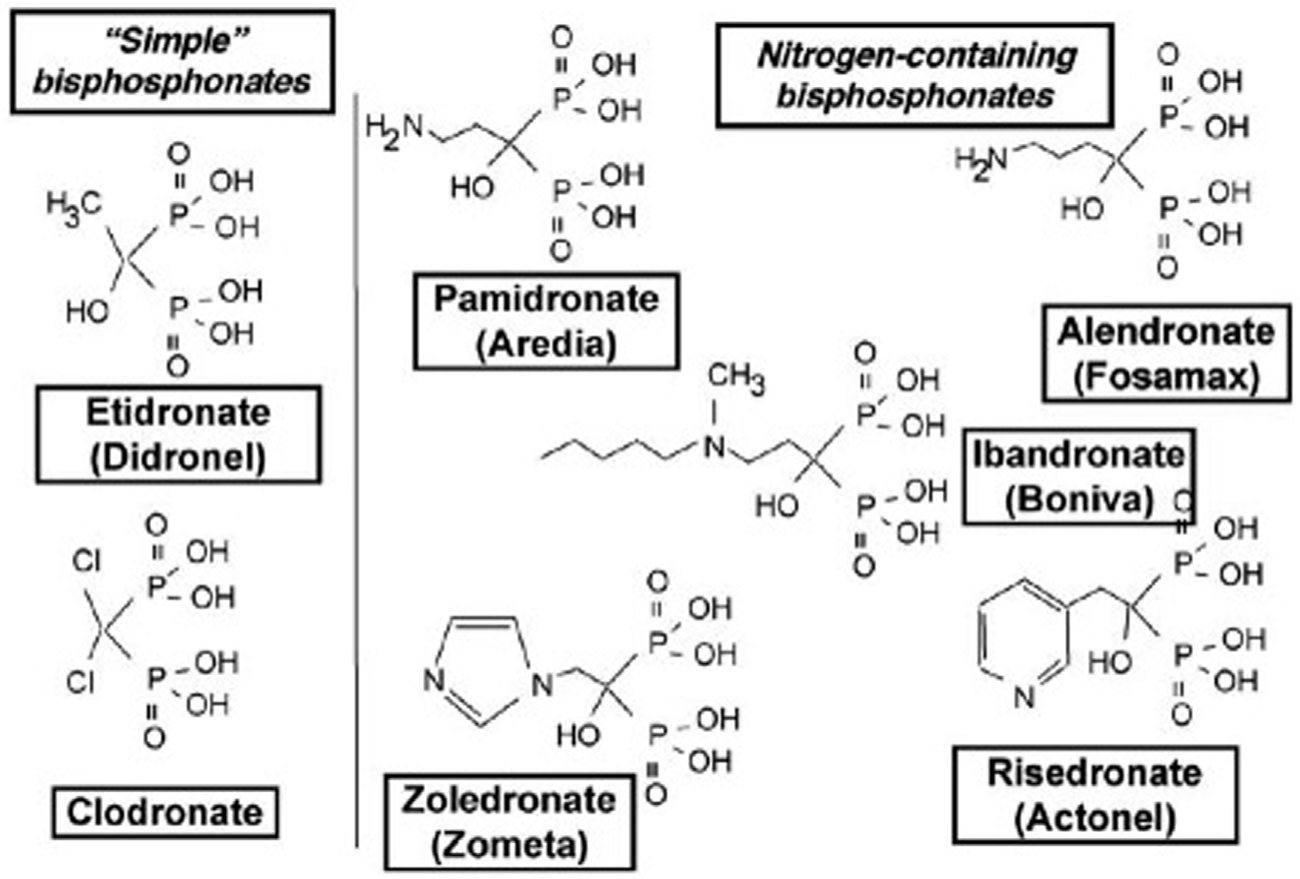
**Schematic representation of the different bisphosphonate (BP) families: simple non-N-containing BPs (etidronate and clodronate) and N-containing BPs (pamidronate, alendronate, idandronate, risedronate, and zoledronate)**.

Bisphosphonates are the synthetic analogs of endogenous pyrophosphate, with a high resistance to protease degradation, and the ability to strongly inhibit bone resorption (33). They are composed of two phosphonate groups. The central oxygen atom in pyrophosphate is replaced by a carbon atom, which allows the substitution of two side groups, one of which is often an hydroxyl group, and the other defines the BP generation (Figure 3). Two main families can therefore be distinguished: nitrogen- and non-nitrogen-containing BPs, which act on osteoclasts by means of different molecular mechanisms. In both cases, the final result – common to both – is the induction of osteoclast apoptosis. BPs act either by inhibiting the recruitment, proliferation, and differentiation of pre-osteoclasts or by impeding the resorptive activity of mature osteoclasts (34–37). Zoledronic acid (ZOL) belongs to the third generation of BPs, which is the most efficient for preventing bone lesions (38–40). As for other nitrogen-containing BPs, ZOL inhibits the farnesyl diphosphate and geranylgeranyl diphosphate synthases, two enzymes involved in the mevalonate pathway necessary for the prenylation of small intracellular GTPases, such as Ras, Rho, or Rac (41). As the prenylation of these GTPases is essential for osteoclast function, their inhibition leads to osteoclast apoptosis as a result of the loss of the survival signal (42–44). Moreover, BPs may also inhibit bone resorption by increasing the production of OPG by human osteoblasts (45). OPG is the decoy receptor of RANKL, which inhibits the RANK/RANKL interaction that is essential for osteoclast differentiation and activation. In addition to the antiresorptive effect of ZOL, it has been shown to induce the death of tumor cell lines, such as myeloma, and breast and prostate carcinoma cells in several preclinical studies (39). It also appears to exert an inhibitory effect on cancer cell invasion and angiogenesis (46).

Despite several side effects reported after long-term treatment with BPs, including osteonecrosis of the jaw, BPs are currently under investigation in postmenopausal bone loss and bone lesions of tumoral origin, such as bone metastases from breast and prostate cancer with variable clinical benefits (47, 48). A significant decrease in bone resorption was observed in these studies but with no unequivocal effects on survival or the occurrence of metastases. The clinical effects of BPs on bone metastases from lung cancer are also discussed, as are their effects on visceral metastases (49–52). The encouraging results reported on bone remodeling, as well as the ability of BPs, and in particular ZOL, to induce tumor cell death *in vitro*, make them good candidates for a therapeutic strategy in primary bone tumors. ZOL may effectively inhibit both bone resorption and tumor proliferation in the vicious cycle, making it more efficient. With regard to primary bone tumors, several studies have already demonstrated the benefits of using ZOL in osteosarcoma (53–56), in particular, promising preclinical results have been reported on survival and tumor growth (56). In this context, ZOL has recently been combined with conventional chemotherapy and surgery for adult and pediatric patients in the French OS2006 phase III randomized clinical trial for osteosarcoma treatment. Following these results, other preclinical and clinical studies demonstrated the beneficial effect of BP treatment in osteosarcoma (57–61).

In Ewing sarcoma, despite improvements to chemotherapy protocols, the survival rate for patients with bone metastases remains very low. In this context, combining ZOL with current conventional chemotherapy may be a promising therapeutic option for both limiting tumor-associated osteolysis and preventing the development of bone metastases, which is currently the main factor for a bad prognosis for this pathology (62). Few fundamental and preclinical studies have demonstrated an anti-tumoral effect for ZOL on Ewing sarcoma cell lines (63, 64). Of these studies, our team has recently shown that ZOL significantly inhibits tumor cell viability by blocking the cell cycle in S-G_2_M phase transition and by promoting caspase-3 activation (65). Using preclinical models of Ewing sarcoma induced in athymic mice by injecting human Ewing sarcoma cells either in bone site or in soft tissue, ZOL alone significantly inhibited tumor development in bone sites, decreasing osteolytic lesions and improving mouse survival (65). On the contrary, the same doses of ZOL had no effect on Ewing sarcoma progression in soft tissue. These results can be explained by the high tropism of BPs for the calcified bone matrix, leading to their elevated concentration in bone tissue and their rapid clearance from blood and soft tissue. These data correlate with other studies on soft tissue tumors or visceral metastases (39). On the other hand, we demonstrated the synergistic effect of a combination of ZOL and ifosfamide, a conventional drug used in Ewing sarcoma clinical protocols, on tumor progression in soft tissue (65). These results correlate with previous studies showing a synergistic effect between BPs and chemotherapeutical agents and demonstrate the great benefit of using ZOL in Ewing sarcoma treatments as a means of reducing the chemotherapy doses and as a consequence, their side effects (58, 66–68).

With regard to invasion and migration, we have already published that treatment with ZOL inhibits Ewing sarcoma cell migration *in vitro* in Boyden chambers and diminishes MMP-2 activity as revealed by zymography (69). In addition, less pulmonary metastases were observed in mice treated with ZOL compared to untreated animals, in a model of spontaneous metastases disseminated from primary Ewing sarcoma induced in bone (69).

For the transfer to clinical practice, one phase II study evaluating the combination of chemotherapy and pamidronate in osteosarcoma patients has demonstrated little impact on patient survival, but has been shown to improve the durability of limb reconstruction (61). In a recently completed phase I study, ZOL combined with conventional multi-drug chemotherapy was safe, but failed to reveal any significant differences in event-free or overall survival in patients with newly diagnosed metastatic osteosarcoma (70). There are three phase II/III trials currently in progress, evaluating the efficacy of ZOL as a single agent or an adjuvant to chemotherapy in localized and metastatic osteosarcoma (NCT00691236 and NCT00470223) and in Ewing sarcoma (NCT00987636).

However, long-term use of BPs may impact bone growth and tooth eruption in young patients. In our laboratory, we have carried out preclinical studies on newborn mice treated or not with ZOL, using a protocol that reproduces the frequency and doses administered in humans. ZOL induces a reversible arrest in bone growth that was also observed in young patients treated with zometa^®^ (71). For tooth eruption, irreversible inhibition was observed (72).

As several side effects have been reported with the clinical use of BPs (49, 50), another approach to decrease bone resorption could therefore be to target RANKL, the main cytokine involved in osteoclast differentiation.

### Anti-RANKL Strategies

Bone remodeling is strongly regulated thanks to the molecular triad OPG–RANKL–RANK (26). The binding of RANKL to its receptor RANK, expressed on the surface of osteoclast precursors, induces osteoclast differentiation *in vitro* in addition to macrophage-colony stimulating factor (M-CSF), suggesting that this differentiation plays an important role in bone biology. In the bone microenvironment, RANKL is produced by bone marrow stromal cells and osteoblasts, while in a bone tumor environment, it can be produced by other cell types, such as fibroblasts, epithelial cells, or T-lymphocytes, in which RANKL appears to be the final effector of osteoclast-mediated bone resorption (26). Cells from many tumor types, including multiple myeloma, prostate cancer, or even human neuroblastoma, can also express RANKL themselves (73–75). Moreover, many of the chemokines, cytokines, hormones, and growth factors produced by tumor cells are able to induce an increase in RANKL expression through PTH-rP, and a decrease in OPG production, thus aggravating the vicious cycle in bone metastases. RANK is one of the signaling molecules associated with worse outcomes in osteosarcoma. High expression of RANKL is associated with reduced survival in osteosarcoma, and it has been reported that osteosarcoma cell lines and biopsies show high expression of functional RANK, suggesting a potential autocrine stimulation of this pathway (76, 77). Inhibition of RANKL using the shRNA strategy reduced motility and anoikis resistance in osteosarcoma cell lines, whereas overexpression of RANK increased OS cell motility without affecting cell proliferation (78). One study reported the preventive effect of siRNA–RANKL on tumor progression when associated with the chemotherapeutic agent ifosfamide in a preclinical model of osteosarcoma (79). For Ewing sarcoma, only a few studies are available, but it seems that these cells express only a low level of RANKL (3). In our case, preliminary preclinical studies evidenced localized but strong expression of RANKL in a paratibial model of Ewing sarcoma induced by an intramuscular injection of human A673 cells in Nude mice (80). The advantages of targeting RANKL have previously been reported in both bone metastases and primary bone tumors and might be a promising target in Ewing sarcoma (73, 74, 81, 82). Several molecules targeting RANKL have already proved their efficiency in other malignant bone pathologies, such as osteosarcoma, and might be a potent therapeutic agent in Ewing sarcoma.

Osteoprotegerin, a member of the TNF receptor super-family, is a ubiquitous secreted homodimeric cytokine able to bind RANKL and then inhibit the RANK/RANKL interaction, as well as any further osteoclast differentiation and activation (83–85). A disruption in the RANKL/OPG ratio in favor of RANKL has been shown to be responsible for severe osteolysis in a tumoral context (86). Accordingly, overexpressing OPG to restore this equilibrium between OPG and RANKL expression appears to be a promising approach for limiting tumor-associated bone lesions. For the first time, our team has shown significant therapeutic benefits of OPG in primary bone tumors. In a preclinical model of osteosarcoma, OPG delivered by non-viral gene transfer effectively inhibited tumor growth and tumor-associated osteolysis, significantly increasing animal survival (81). Several studies have tested OPG overexpression in OS and Ewing sarcoma preclinical models with promising results, especially in osteosarcoma (81). Moreover, despite its clinical efficiency in preventing osteolytic lesions, a major issue for OPG-Fc administration as an adjuvant therapeutic agent in a tumor context is its ability to inhibit the apoptosis induced by TNF-related apoptosis inducing ligand (TRAIL) (87). The dual effect of OPG may inhibit TRAIL-induced apoptosis of tumor cells, a natural mechanism for preventing tumor development (88). In addition, TRAIL’s ability to both induce apoptosis in sensitive Ewing Sarcoma cell lines and prevent tumor development has already been demonstrated *in vitro* by Wietzerbin’s team and *in vivo* by our team in a preclinical model induced by intratibial injection of Ewing sarcoma cells in nude mice (89, 90). To avoid the potential protumoral effect of OPG, the recombinant protein RANK-Fc, the soluble form of RANK, could be used in Ewing sarcoma to block RANKL activity. RANK-Fc is unable to bind TRAIL, and its efficacy has already been demonstrated in preventing tumor-associated osteolysis and, indirectly, tumor growth in preclinical models of bone metastases, such as prostate, lung, and breast cancer (91–93). Our team also showed how RANK-Fc, when delivered by non-viral gene transfer, is able to prevent osteolytic lesions and tumor development, thus inducing an increase in animal survival in a preclinical rodent model of osteosarcoma (82). The same efficacy can be expected in Ewing sarcoma but remains to be tested. For clinical transfer, denosumab is a monoclonal antibody specific for human RANKL, which was initially developed to treat osteoporosis (94). It was then used for painful bone metastases with effective results (95–98). It was subsequently found to also be effective for giant cell tumor of bone, a benign but destructive neoplasm with severe osteolytic lesions, in which transformed mononuclear cells secrete high levels of RANKL, causing osteoclast hyperactivity (99).

## Targeting Other Aspects of the Bone Microenvironment in Ewing Sarcoma

**Figure 4:**
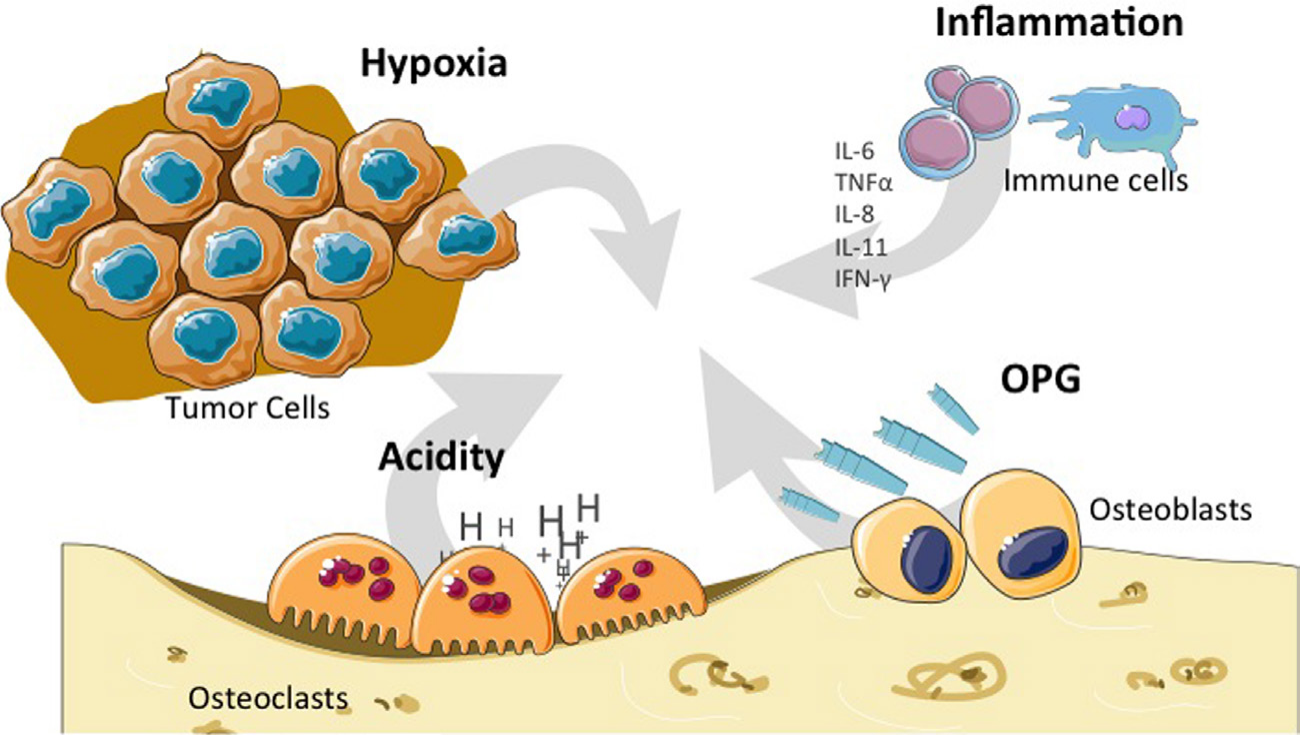
**Particular bone microenvironment that may affect tumor initiation, progression, or dissemination**. Bone microenvironment is characterized by high hypoxia, high acidity, OPG release, and the influence of the cytokines (IL-6, IL-8, IL-11, and TNF-α) produced by cells from the immune system. IL, interleukin; TNF, tumor necrosis factor; OPG, osteoprotegerin.

Besides bone cells themselves, the tumor microenvironment of primary bone tumors provides factors that are favorable for tumor initiation, progression, therapy resistance, or metastatic dissemination. Of the different constituents or aspects of this peculiar microenvironment, special attention has been paid to hypoxia, escape from the immune system, angiogenesis, growth factors from the microenvironment, and modification of the microenvironment itself by therapeutic agents that may interfere with tumor progression (Figure 4).

*Hypoxia* is an important condition in the tumor cell microenvironment associated with a more aggressive phenotype and poor prognosis of many cancers in adults. For example, intratumoral hypoxia is a common finding in breast cancer associated with a significantly increased risk of metastasis and patient mortality (100). Hypoxia-inducible factors activate the transcription of a large battery of genes encoding proteins that promote primary tumor vascularization and growth, stromal cell recruitment, extracellular matrix remodeling, pre-metastatic niche formation, cell motility, local tissue invasion, extravasation at sites of metastasis, and maintenance of the cancer stem cell phenotype that is required to generate secondary tumors. It is also known that severe and long-lasting hypoxia results in necrosis, thus being correlated with unfavorable outcome. Concerning Ewing sarcoma, a clinical study previously reported a strong correlation between the presence and the amount of necrotic areas in the tumor with the risk of metastases (101). In addition, Aryee et al. reported that HIF-1α expression was detectable in 18/28 primary tumors from the Ewing sarcoma family and that EWS-FLI1 was up-regulated in a HIF-1α-dependent manner (102). In addition, this study revealed that hypoxia stimulated the invasiveness and soft agar colony formation of Ewing sarcoma cells *in vitro*. Further studies suggest that EWS-FLI1 regulation in an hypoxic environment may occur at the posttranscriptional level, which is supported by the observation that HIF-1α-activated genes, such as VEGF, Aldolase-C, GLUT-1, CA9, and IGFBP3, were increased under hypoxia, whereas EWS-FLI1 RNA expression remained unchanged (103). It is also suggested that hypoxia increases Ewing sarcoma malignancy through enhancing invasive and colony-formation capacities. Furthermore, it could be proposed that hypoxia may contribute to the aggressive metastatic behavior of Ewing sarcoma, as HIF-1α and EWS-FLI1 may function together in both synergistic and antagonistic cross-talk under hypoxia conditions. Therefore, drugs that target hypoxia need to be tested in Ewing sarcoma models.

*Crosstalk between the bone niche and the immune system*, known as “osteoimmunology,” has been suggested as being a potential target for bone tumor treatment. There is a well-recognized link between bone constituents and the immune system, leading to recent efforts to elucidate the functions of molecules expressed in both bone and immune cells. A recent review nicely describes the complexity of the interaction between the skeletal and immune systems, suggesting that their interdependency needs to be taken into consideration when designing therapeutic approaches for either of the two systems (31). For example, denosumab, which was originally used to specifically target bone resorption, is now under evaluation for its effect on the long-term immune response. As both the bone and immune systems are often disrupted in cancer, they may be crucial in regulating tumor growth and progression. Certain therapies, such as BPs and RANKL-targeted drugs that aim to reduce pathological osteolysis in cancer, may interact with the immune system, thus providing favorable effects on survival. Another interesting publication reported that dynamic tumor–host immune interactions within the tumor microenvironment may polarize immune responses *in situ*, influencing tumor development and/or progression (104). They studied the nature of tumor–host immune interactions within the Ewing sarcoma microenvironment, analyzing the presence and spatial distribution of infiltrating CD8(+)(/)CD4+ T-lymphocytes in therapy-naive Ewing sarcoma. They observed that tumor-infiltrating T-cells contained significantly higher percentages of CD8(+) T-lymphocytes than stroma-infiltrating cells, suggesting preferential migration of this type of T-cell into tumor areas. Their results indicated that an inflammatory immune microenvironment with high expression of type 1-associated chemokines may be critical for the recruitment of CD8(+) T-lymphocytes expressing the corresponding chemokine receptors. The observed impact of tumor-infiltrating CD8(+) T-lymphocytes is consistent with there being a role for adaptive anti-tumor immunity in preventing Ewing sarcoma from progressing. Recognizing the merits and exploitation/induction of an inflammatory microenvironment may thus improve the efficacy of natural responses against, and (adoptive) immunotherapeutic approaches for, Ewing sarcoma.

*With regard to angiogenesis*, VEGF-165 expression in the tumor microenvironment has been shown to influence the differentiation of bone marrow-derived pericytes, which play a part in the vasculature of Ewing sarcoma (105). One year later, the same team demonstrated that VEGF-165 contributed to the osteolytic process in Ewing sarcoma by upregulating RANKL (106). They showed that VEGF-165, together with EWS-FLI1, increased RANKL promoter activity. This increase in *RANKL* gene expression in the bone marrow microenvironment during the metastatic process may be involved in tumor-induced bone osteolysis.

Other *growth factors* present in the bone microenvironment, such as basic FGF, may play a part in tumor progression as they enhance cell motility and invasion of the Ewing sarcoma family of tumors by activating the FGFR1–PI3K–Rac1 pathway (107). The authors therefore conclude that the bFGF–FGFR1–PI3K–Rac1 pathway in the bone microenvironment may have a significant role in the invasion and metastasis of the Ewing sarcoma family of tumors.

Conversely, *therapeutic agents*, such as ZOL, are able to modify the bone microenvironment surrounding primary or disseminated tumor cells, as has been reported in breast cancer recurrence in bone (108). Treatment of mice with ZOL induced a rapid increase in trabecular bone volume versus controls, which was reflected by a significant reduction in osteoclast and osteoblast numbers per millimeter in trabecular bone, and reduced bone marker levels in serum. Pre-treatment with ZOL caused an accumulation of extracellular matrix in the growth plate associated with a trend for preferential homing of tumor cells to osteoblast-rich areas of bone, but without affecting the total number of tumor cells. The number of circulating tumor cells was reduced in ZOL-treated animals. Although this study concerns breast cancer, osteoblasts may be key components in the bone metastasis/tumor niche, and therefore a potential therapeutic target, at least in breast cancer. This hypothesis therefore needs to be studied extensively in primary bone tumors, including Ewing sarcoma.

## Bone Microenvironment Modeling in Ewing Sarcoma

As the microenvironment, and especially the bone tumor microenvironment, can both inhibit and facilitate tumor growth and metastatic dissemination, better modelization of the tumor bone niche is needed to characterize tumor cell–stroma interaction in depth. It has been shown that osteoblasts, osteoclasts, fibroblasts, myeloid cells, and mesenchymal stem cells (MSCs) play essential roles in primary tumor growth and metastasis (109, 110). However, current *in vitro* approaches are far from replicating the native *in vivo* milieu in which tumors develop, a necessary condition for advancing cancer research and translating new therapies into clinical practice. Most preclinical anti-neoplastic drug testing is still carried out on conventional 2D cell culture systems. Although these systems mimic some of the phenotypic traits observed clinically, they are limited in their ability to model the full range of microenvironmental interactions, such as 3D cell–cell and cell–extracellular matrix interactions. Several teams have thus established *ex vivo* 3D bone tumor models that closely mimic the morphology, growth kinetics, and protein expression profile of human tumors, including Ewing sarcoma (111–113). For example, Ewing sarcoma cells cultured in porous 3D electrospun poly(ϵ-caprolactone) scaffolds were not only more resistant to traditional cytotoxic drugs than cells in 2D monolayer cultures but also exhibited remarkable differences in the expression pattern of the IGF-1R/mTOR pathway (111). This 3D model of the bone microenvironment may therefore have broad applicability for mechanical studies of bone sarcomas and shows the potential for increasing preclinical evaluation of anti-neoplastic drug candidates for these malignancies. In the same way, Villasante et al. described a bioengineered model of human Ewing sarcoma that mimics the native bone tumor niche with high biological fidelity (113). In this model, cancer cells that have lost their transcriptional profiles after monolayer culture re-express genes related to focal adhesion and cancer pathways. The bioengineered model recovers the original hypoxic and glycolytic tumor phenotype and makes possible re-expression of angiogenic and vasculogenic mimicry features that favor tumor adaptation. Differentially expressed genes between the monolayer cell culture and native tumor environment may thus be potential therapeutic targets that could be explored using the bioengineered tumor model.

In addition, Ludwig’s team has highlighted a number of innovative methods used to fabricate biomimetic Ewing sarcoma, including both the surrounding cellular milieu and the extracellular matrix. These methods suggest potential applications for advancing our understanding of the biology of Ewing sarcoma, preclinical drug testing, and personalized medicine (112).

Finally, it appears that the bone microenvironment should be modelized in order to analyze the response of bone tumor cells to drug screening under optimal conditions. Currently, few preclinical models of bone cancer, and particularly Ewing sarcoma, mirror the site of the disease in patients, as they are mostly subcutaneous or intramuscular xenografts (114).

For metastasis in Ewing sarcoma, intravenous models induced in non-obese diabetic/severe combined immunodeficient (NSG) mice showed a pattern of disease spread similar to that found in patients, but only 23% of the experimental mice developed assessable bone metastases (115). It is therefore preferable to develop orthotopic models that involve direct injection of Ewing sarcoma cells at the clinically relevant site, i.e., intrafemoral. This type of injection in immunocompromized mice provides a technically feasible and reproducible approach, resulting in tumors that are detectable by palpation or *in vivo* imaging, and that closely resemble those observed in patients (116). The importance of such orthotopic models for testing potential new drugs at the preclinical level was emphasized in the study by Odri et al. (65), comparing how tumor progression responds to ZOL in two models of Ewing sarcoma: one induced by tumor cell injection in the medullar cavity of tibia and the other with initial progression in soft tissue (65). ZOL significantly inhibited Ewing sarcoma cell progression only in the intratibial model and showed no effect in the soft tissue. These results strongly suggest the importance of considering the complete bone microenvironment when testing new drugs, especially in the case of bone tumors, such as Ewing sarcoma. Recently, Vormoor et al. also developed an interesting preclinical orthotopic model of Ewing sarcoma in NSG mice, reproducing the biology of the tumor–bone interactions observed in human disease (117). In this model, the Ewing sarcoma cells have been modified allowing *in vivo* monitoring of disease progression (115). The authors therefore demonstrated the utility of small animal bioimaging for tracking disease progression, making this model a useful assay for preclinical drug testing.

## Conclusion – Perspectives

Despite improvements in poly-chemotherapy combinations and surgical approaches preserving limbs from amputation, one group of Ewing sarcoma patients still remains at high risk, with poor survival rates. These patients present with metastatic disease at diagnosis or respond poorly to chemotherapy due to acquired resistance. New therapeutic options are thus needed. Given the growing interest in the microenvironment and its recognized involvement in cancer initiation and progression, it is relevant to propose therapeutic strategies that target molecular and/or cellular protagonists of the bone tumor microenvironment in the case of Ewing sarcoma.

Most on-going studies focus on bone cells, especially osteoclasts, either by directly targeting them or inhibiting RANKL, the main cytokine involved in osteoclast activation. These strategies (BPs, anti-RANKL: denosumab) could be proposed not only to target the bone component of the primary tumor but also to target bone/bone marrow metastases, the worst prognosis factor for Ewing sarcoma patients, as confirmed in the R3 arm of the latest EuroEWING99 trial (survival rate of <20% at 5 years). However, as expected, these strategies have no effect in preclinical models of pulmonary metastases, which remains the main cause of mortality in Ewing sarcoma patients (the prognosis for patients with lung-only metastases is 30% survival at 5 years). However, the strategies could be proposed for pulmonary metastatic patients or patients with soft tissue Ewing sarcoma if they are in synergy with current or targeted therapies, as suggested by our preclinical studies combining ZOL with ifosfamide (65).

## Conflict of Interest Statement

The authors declare that the research was conducted in the absence of any commercial or financial relationships that could be construed as a potential conflict of interest.
